# Comparative effectiveness of propylene glycol–based health programs for controlling ketosis and improving performance in dairy cows

**DOI:** 10.14202/vetworld.2026.2051-2066

**Published:** 2026-05-18

**Authors:** Xin-Zhuo Yu, Lian-Ying Wang, Xiao-Chen Jia, Ke-Ya Yu, Sheng-Yu Han, Guang Shao, Yu-Xi Song, Cheng Xia

**Affiliations:** 1 Animal Metabolic Disease Lab, College of Animal Science and Technology, Heilongjiang Bayi Agricultural University, Daqing, China; 2 Daqing Agricultural and Rural Social Undertakings Service Center, Daqing, China; 3 Heilongjiang Mudanjiang Agricultural Reclamation Jiangjun Dairy Cow Breeding Professional Cooperative, Jixi, China; 4 Agricultural Development Department of Beidahuang Group Heilongjiang 8510 Farm Co., Ltd., Jixi, China; 5 Heilongjiang Mudanjiang Agricultural Reclamation Qianmu Dairy Farm, Jixi, China; 6 Branch of Animal Husbandry and Veterinary, Heilongjiang Academy of Agricultural Sciences, Qiqihar, China

**Keywords:** body condition score, dairy cows, ketosis, metabolic disorder, negative energy balance, production performance, propylene glycol, transition period

## Abstract

**Background and Aim:**

Ketosis is a prevalent metabolic disorder in dairy cows during the transition period, resulting in reduced productivity, impaired reproductive performance, and significant economic losses. Propylene glycol (PG) is widely used as a gluconeogenic precursor to mitigate negative energy balance and control ketosis. However, variations in farm-level health programs lead to inconsistent outcomes. This study aimed to comparatively evaluate the effectiveness of different PG-based health programs implemented across multiple commercial dairy farms.

**Materials and Methods:**

A multi-farm observational study was conducted on 480 multiparous Holstein cows from four commercial dairy farms in Heilongjiang Province, China. Data were collected at six time points from 7 days prepartum to 80–100 days postpartum. Parameters assessed included body condition score (BCS), milk yield, reproductive performance, disease incidence, and blood biochemical indicators such as glucose, β-hydroxybutyric acid, and insulin. Statistical analyses were performed using linear mixed models and Chi-square tests, with significance declared at p < 0.05.

**Results:**

Significant differences were observed among farms in metabolic status, production performance, and disease incidence. Farm A, which applied a targeted PG administration strategy based on ketosis diagnosis, maintained optimal BCS (~3.4), achieved the highest milk yield (46.3 kg/d at 80–100 days postpartum), and exhibited stable glucose and insulin concentrations. The incidence of ketosis in Farm A (28.9%) was lower than that in Farm D (>35%), which showed poorer metabolic control, higher disease incidence, and reduced reproductive performance. Farm B demonstrated the lowest ketosis incidence (20.0%) but relied on blanket PG administration. Economic analysis revealed that Farm A achieved the highest total economic benefit (¥166,823.69).

**Conclusion:**

Targeted and time-specific PG administration is more effective than blanket or inconsistent strategies in controlling ketosis and improving productivity in dairy cows. The PG health program implemented in Farm A represents a practical and economically efficient model for commercial dairy systems. Further controlled studies are required to validate these findings and optimize long-term PG application strategies.

## INTRODUCTION

The transition period is defined as the period from 3 weeks prepartum to 3 weeks postpartum in cows [1]. During this period, cows undergo significant physiological changes associated with pregnancy, calving, and lactation, which can readily result in a range of postpartum diseases, the most prevalent of which is ketosis [2, 3]. As the energy requirements of dairy cows increase, their feed intake cannot meet the body’s needs, resulting in insufficient carbohydrate intake and glucose storage, leading to a negative energy balance (NEB) [4, 5]. To compensate for this shortage of energy, the body initiates fat mobilization as a surrogate, leading to an increase in the concentration of non-esterified fatty acids (NEFA) in the blood. Excess NEFA enters the ketone body synthesis pathway and produces high concentrations of β-hydroxybutyric acid (BHBA), acetoacetate, and acetone, leading to ketosis [6, 7]. Studies show that 15% of dairy cows worldwide suffer from ketosis, and this proportion is still on the rise [8, 9]. Ketosis is divided into clinical ketosis and subclinical ketosis. Clinical ketosis usually presents a series of symptoms, such as decreased appetite, weight loss, reduced milk yield, and poor milk quality [10, 11]. Failure to treat ketosis in a timely manner results in decreased body resistance and secondary infections, increasing the difficulty and cost of treatment [12]. A scientific and reasonable health care plan can reduce the incidence of ketosis and other postpartum diseases in dairy cows. Although various intervention measures are available to reduce the occurrence of ketosis, the disease still poses a significant threat to modern dairy farming. Given the limitations of existing farm health programs, propylene glycol (PG), as a common treatment for ketosis, warrants further evaluation in diverse on-farm settings.

PG is widely used to treat and prevent ketosis in dairy cows [13, 14]. Feeding PG can increase lactation and decrease postpartum morbidity in dairy cows [15, 16]. As a gluconeogenic precursor, PG has been shown to promote glucogenesis and mitigate NEB [17]. Oral PG is rapidly absorbed, increases plasma glucose concentration, significantly reduces plasma BHBA concentration, regulates blood metabolism, improves energy balance, and enhances reproductive performance in cows [18, 19]. Several studies have demonstrated that PG effectively treats ketosis in dairy cows, and that different doses of PG and associated supplements exert variable effects on treatment outcomes [20, 21].

Despite extensive research on ketosis in dairy cows, most existing studies have primarily focused on controlled experimental conditions, single-herd randomized trials, or short-term intervention strategies using PG. While these studies have demonstrated the efficacy of PG in improving metabolic status and reducing BHBA concentrations, their applicability under diverse commercial farm conditions remains limited. In particular, there is a lack of comparative evidence evaluating different PG-based health programs implemented across multiple farms with varying management practices, nutritional strategies, and treatment protocols. Furthermore, current literature provides insufficient insight into how differences in timing, dosage, and administration strategy (targeted versus blanket treatment) influence production performance, disease incidence, reproductive outcomes, and economic returns simultaneously. Additionally, few studies integrate metabolic, clinical, and economic indicators within a single analytical framework, which is essential for practical decision-making in large-scale dairy operations. Therefore, a critical knowledge gap exists in identifying the most effective and economically sustainable PG-based health program under real-world, multi-farm conditions.

The present study aimed to comparatively evaluate the effectiveness of different PG-based health programs implemented across multiple commercial dairy farms during the transition period. Specifically, this study sought to (i) assess the impact of varying PG administration strategies on body condition score (BCS), milk yield, and key metabolic indicators, including BHBA, glucose, and insulin; (ii) determine the influence of these programs on disease incidence and reproductive performance; and (iii) analyze the associated economic outcomes under practical farm conditions. By integrating production, metabolic, clinical, and economic data, this study aimed to identify an optimal PG health program that enhances productivity, reduces ketosis incidence, and improves overall herd performance. The findings are expected to provide scientifically grounded and practically applicable recommendations for optimizing ketosis management strategies in commercial dairy systems.

## MATERIALS AND METHODS

### Ethical approval

Ethical approval for this study was obtained from the Ethical Committee of Heilongjiang Bayi Agricultural University (Approval No. DWKJXY2023057; approved on 1 August 2023). All procedures involving animals were conducted in accordance with the institutional guidelines for the care and use of animals in research, as well as relevant national regulations governing animal welfare.

The study was carried out on commercial dairy farms under standard management and feeding practices, and no invasive or harmful procedures were performed specifically for research purposes. Data collection was limited to diet composition analysis and nutrient evaluation, along with routine production-related observations. Animal handling, where required, was performed by trained personnel following standard husbandry practices to minimize stress and ensure animal welfare.

Permission was obtained from the owners/managers of all participating farms prior to the commencement of the study. The research adhered to the principles of animal welfare, including reduction of unnecessary intervention and avoidance of distress, ensuring that all activities were conducted under normal farm conditions without disrupting routine management practices.

### Study period and location

This animal study was conducted between November 2024 and February 2025 on four commercial Holstein dairy farms located in Heilongjiang Province, Northeast China. The farms operated under intensive housed systems with similar herd sizes (more than 3,000 milking cows) and used freestall barns with concrete flooring and cubicles.

### Study design and farm selection

The four farms were purposively selected because they are large-scale dairy farms in Heilongjiang Province, with a large number of dairy cows, and represent a significant sample. Additionally, they already implemented different PG-based postpartum health programs for ketosis prevention and treatment as part of their routine veterinary management. No farm used exactly the same protocol, allowing direct comparison of real-world implementation variations. Farms were not randomized and no control (no-PG) group was included, as this was an observational comparison of existing on-farm practices.

### Animals and inclusion criteria

A total of 480 multiparous Holstein cows were initially enrolled, with 120 cows selected from each of the four farms as a fixed cohort. At each of six sampling points (–7 d prepartum, 0 d [calving day], +7 d, +21 d, +50–60 d, +80–100 d postpartum), 20 cows were randomly selected from this cohort (n = 20 × 4 farms × 6 time points = 480 observations). Cows were treated as a random effect nested within farm.

Cows enrolled in all farms were highly similar in parity, age, and BCS at the initiation of the study, with strict inclusion and exclusion criteria applied uniformly across farms. Inclusion criteria: Holstein cows of similar age, BCS, and parity [41.26 ± 0.74 months of age, 3.11 ± 0.02 of BCS] multiparous cows (≥2nd lactation, 2.33 ± 0.06 of parity), clinically healthy at enrollment (no obvious systemic disease), and due to calve within ±7 days of the sampling window. Exclusion criteria: cows with severe clinical mastitis, metritis, displaced abomasum, or lameness score >3 at enrollment. Furthermore, the 120 cows selected from each farm were under continuous long-term observation, with no mortality or culling during the entire experimental period.

### Dietary management

All the farms supply total mixed ration (TMR) twice a day, at 7:00 am and 1:00 pm after milking. The composition and nutrient levels of the basal diet are presented in Table 1. Rations were formulated to meet or exceed NRC (2001) nutrient requirements for lactating Holstein cows producing 35–45 kg milk/d [22]. Feed samples were collected weekly and analyzed for dry matter, crude protein, neutral detergent fiber (NDF), acid detergent fiber (ADF), starch, and net energy by a commercial laboratory. The quantification of DM (at 105°C for 4 h) and crude protein (CP) in the feed was performed following AOAC (2006) guidelines, utilizing methods 934.01 for DM and 990.03 for CP [23]. The starch content in the feed was analyzed according to the respective China National Standard (GB/T 20194-2018) [24]. NDF contents were measured using the F57 fiber bag (25 mm porosity) technique (ANKOM Technology, Fairport, NY, USA) in accordance with China National Standard (GB/T 20806-2022) [25]. During the NDF analysis, thermostable α-amylase and sodium sulfite were employed to remove the interference of starch and protein, respectively.

**Table 1:** Diet composition and nutritional levels of dairy cow from four farms.

Item	Farm A	Farm B	Farm C	Farm D
Diet composition, % of DM				
Water	15.12	15.01	14.89	14.48
Alfalfa	11.10	10.45	10.56	10.86
Ensiling	38.65	39.2	40.1	39.5
Whole cotton seed	3.1	2.6	2.7	2.9
Concentrates	18.7	18.2	19.0	19.1
Molasses	4.01	3.34	3.21	3.29
Flaked corn	10.21	9.75	9.91	9.87
Nutrient levels, % of DM				
Net milk production (Mcal/kg)	1.72	1.83	1.73	1.61
Crude protein (%)	16.48	15.79	16.21	16.53
Acid detergent fiber (%)	17.31	17.35	18.31	17.42
Neutral detergent fiber (%)	29.52	28.31	31.21	30.13
Starch (%)	28.51	28.11	29.64	29.61
Vitamin E (IU/kg)	59.63	58.91	59.32	60.05
Selenium (mg/kg DM)	0.67	0.72	0.68	0.66

### PG health programs

During the experiment, dairy cows in each farm were densely raised in cattle sheds with the same strict hygiene conditions and free movement, receiving the same standardized care and being able to drink water freely. Throughout the experimental period, dairy cows from four farms were initially administered fluid nutrient supplements to ameliorate their nutritional status; subsequent steps involved clinical screening for ketosis and implementation of a PG health program. The detailed formulation of the nutritional supplement is presented in Table 2.

**Table 2:** Nutritional supplement ingredients.

Item	Content
Propylene glycol (mL)	500
Yeast (g)	100
Potassium bicarbonate (g)	100
Magnesium sulfate (g)	120
Salt (g)	50
Sodium bicarbonate (g)	100
Selenium yeast (g)	10

The above nutritional supplement components were incorporated into 20 kg of water and administered via an esophageal drenching tube early in the morning postpartum.

Each farm applied its established PG-based health programs without modification for the study. Programs differed in three key aspects: (1) the timing of first PG administration, (2) whether PG was given prophylactically or only to diagnosed cases, and (3) the number of consecutive treatment days. Details are presented in Table 3. For dairy cows in Farm A, 500 mL of the nutritional supplement was administered via oral drenching on the day of calving; ketosis screening was subsequently performed at 7 and 14 days postpartum, and cows diagnosed with ketosis were given 500 mL of PG daily for 2 consecutive days. In Farm B, cows received co-administration of 500 mL nutritional supplement and 500 mL PG via oral drenching on the day of calving; ketosis detection was carried out at 7 and 14 days postpartum, with a single 500 mL dose of PG administered to all cows diagnosed with ketosis. For Farm C, 500 mL of the nutritional supplement was orally drenched to cows on the day of calving, followed by ketosis screening at 7 and 14 days postpartum. Cows identified with ketosis at 7 days postpartum were given 500 mL of PG daily for 2 consecutive days, while those diagnosed at 14 days postpartum received a single 500 mL dose of PG. In Farm D, cows were administered 500 mL of the nutritional supplement via oral drenching on the day of calving; ketosis detection was conducted at 7 and 14 days postpartum, and a single 500 mL dose of PG was given to any cow diagnosed with ketosis. During the oral administration process, all cows demonstrated good tolerance to PG administration, with no adverse clinical signs observed. Treatment acceptance was high, and cows completed the drenching procedure without resistance.

**Table 3:** Propylene glycol health program of dairy cows on four farms.

Index	Item	Farm A	Farm B	Farm C	Farm D
Oral drenching (mL)	0 d	Nutritional supplement 500	Nutritional supplement 500 / PG 500	Nutritional supplement 500	Nutritional supplement 500
	7 d	PG 500*2d	PG 500	PG 500*2d	PG 500
	14 d	PG 500*2d	PG 500	PG 500	PG 500

### Ketosis screening and disease definition

On the day of calving and 14 days postpartum, the specific enzyme-linked immunosorbent assay kit for cattle (Shanghai Enzyme-Linked Biotechnology Co., Ltd., Shanghai, China) was used to measure BHBA in the experimental cow’s serum, thereby screening for ketosis. Subclinical ketosis was defined as BHBA ≥1.20 mmol/L, and clinical ketosis was diagnosed when BHBA ≥3.0 mmol/L [26] and at least two of the following signs were present: reduced feed intake, decreased milk yield, dullness/lethargy, or detectable abnormal odor on breath. All measurements were performed by trained farm veterinarians blinded to farm protocol allocation during sample collection.

The definitions of other diseases in the experiment are as follows. A retained placenta is defined as the failure of the placenta to be expelled within 24 h after calving [27]. When a cow experiences a sharp decline in milk yield one week before onset, accompanied by a significant decrease in rumination and activity levels, and percussion and auscultation reveal a high-pitched “pinging”, it is diagnosed with true displaced abomasum [28]. When a cow shows signs of miscarriage after confirmation of pregnancy, comes into heat again after confirmation of pregnancy, or is found to have failed to conceive upon re-confirmation of pregnancy, this is defined as a miscarriage [29]. When the somatic cell count in cow’s milk is ≥ 200,000 cells/mL, it is diagnosed as mastitis [30]. The characteristics of metritis in dairy cows include foul-smelling, watery, reddish-brown discharge, with or without fever [31]. Dystocia is usually defined as any delivery process that requires assistance [32]. Clinical milk fever in dairy cows is defined as a serum calcium concentration below 2.0 mmol/L, accompanied by clinical symptoms such as recumbency [33]. Hoof disease is defined as a condition often accompanied by increased hoof temperature, pain, and subsequent lameness [34].

### Data collection

The following variables were recorded: (1) The BCS of dairy cows is assessed on a 5-point scale (1 = emaciated, 5 = obese) [35], assessed by two trained veterinarians (average used). (2) The daily milk yield (kg) of dairy cows is recorded by an electronic milk meter and is recorded using specific software (Afifarm, Afimilk, Kibbutz Afikim, 1514800, Israel) to record age, parity, milk yield, and disease incidence of the cows. (3) The milk components (including fat, protein, urea nitrogen) are measured monthly DHI laboratory. (4) The disease events in dairy cows (including ketosis, retained placenta, displaced abomasum, abortion, mastitis, metritis, dystocia, milk fever, and hoof disease) are diagnosed and recorded by veterinarians. (5) The observation of the reproductive status of dairy cows, including the number of services, calving to first service interval, calving to first estrus interval, calving interval, and conception rate (calculated as total conception rate × conception rate at estrus × [1 - empty rate]).

### Blood sampling and laboratory analyses

Blood (10 mL) was collected from the coccygeal vein before morning feeding into plain serum tubes. Samples were centrifuged at 4,000 × *g* for 10 min at 4°C within 2 h of collection. Serum was stored at -80°C until analysis. Glucose, calcium, phosphorus, magnesium, and potassium were measured using an automated biochemistry analyzer (Mindray BS-830S) using commercial kits (Nanjing Jiancheng Bioengineering Institute Co., Ltd, Nanjing, China). BHBA and insulin were detected using bovine-specific ELISA kits (Shanghai Enzyme-linked Biotechnology Co., Ltd., Shanghai, China). Intra- and inter-assay CV were <10% for all assays.

### Statistical analysis

Data were analyzed using IBM SPSS 26.0 software (SPSS Inc., Chicago, IL, USA). A linear mixed model was used to analyze continuous variables (BCS, milk yield, blood metabolites), with farm, time point, and their interaction as fixed effects, and cow nested within farm as a random effect. Tukey’s Honest Significant Difference post hoc test was applied to identify specific inter-group differences with adjustment for multiple comparisons. Categorical variables (disease incidence) were compared using Chi-square tests; odds ratios (OR) and 95% confidence intervals (95% CI) were computed. Significance was declared at p < 0.05; extremely significant was set at p < 0.01, and trends at 0.05 ≤ p < 0.10. Results were presented as means ± SEM.

The calculation method for the economic efficiency of cattle farms is as follows:

Economic benefit of ketotic cows = total × morbidity rate × (milk yield × milk price - cost).

Economic benefit of healthy cows = total × healthy rate × (milk yield × milk price - cost).

Total economic benefit = economic benefit of sick cows + economic benefit of healthy cows - cost of PG feeding.

The price of milk was 3.21 ¥/kg.

## RESULTS

### Effects of different PG health program on BCS and milk yield of dairy cows in four farms

**Figure 1:**
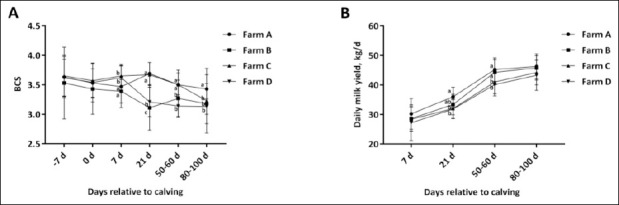
Effects of different PG health program on milk yield and BCS of dairy cows in four farms. n = 20 per farm at each time point. (a) BCS, (b) milk yield. Significant differences (p < 0.05) are indicated by different lowercase letters.

At 7 days postpartum, the BCS of cows in Farm C (3.65 ± 0.35) and Farm D (3.62 ± 0.31) was significantly higher than that of cows in the other two farms (p < 0.05). At 21 days postpartum and from 50 to 60 days postpartum, the BCS of cows in Farm A and Farm C was significantly higher than that of cows in the other two farms (p < 0.05). From 80 to 100 days postpartum, the BCS of cows in Farm A (3.43 ± 0.24) was significantly higher than that of cows in the other three farms (p < 0.05) (Table 4, Figure 1a). At 21 days postpartum and from 50 to 60 days postpartum, cows in Farm A and Farm B had significantly higher milk yield than those in the other two farms (p < 0.05) (Table 5, Figure 1b).

**Table 4:** BCS of dairy cows in four farms.

Time (d)	Farm A	Farm B	Farm C	Farm D
–7	3.63 ± 0.35	3.53 ± 0.61	3.65 ± 0.35	3.62 ± 0.31
0	3.53 ± 0.24	3.43 ± 0.43	3.57 ± 0.29	3.54 ± 0.33
7	3.47 ± 0.36ᵃ	3.39 ± 0.20ᵃ	3.65 ± 0.17ᵇ	3.62 ± 0.23ᵇ
21	3.69 ± 0.19ᵃ	3.11 ± 0.38ᶜ	3.67 ± 0.21ᵃ	3.21 ± 0.25ᵇ
50-60	3.5 ± 0.25ᵃ	3.27 ± 0.31ᵇ	3.5 ± 0.2ᵃ	3.14 ± 0.19ᵇ
80-100	3.43 ± 0.24ᵃ	3.18 ± 0.34ᵇ	3.23 ± 0.55ᵇ	3.13 ± 0.13ᵇ

Significant differences (p < 0.05) are indicated by different lowercase letters.

**Table 5:** Daily milk yield of dairy cows in four farms.

Time (d)	Farm A	Farm B	Farm C	Farm D
7	30.2 ± 5.2	28.6 ± 4.2	28.4 ± 4.0	27.2 ± 6.1
21	36.0 ± 3.2ᵃ	33.4 ± 3.5ᵃᵇ	32.0 ± 3.3ᵇ	31.8 ± 3.2ᵇ
50-60	45.2 ± 4.0ᵃ	44.3 ± 4.2ᵃ	41.0 ± 4.0ᵇ	40.1 ± 3.8ᵇ
80-100	46.3 ± 4.3	45.8 ± 4.1	44.2 ± 4.2	43.3 ± 5.1

Significant differences (p < 0.05) are indicated by different lowercase letters.

### Effects of different PG health program on milk composition of dairy cows in four farms

From 50 to 60 days postpartum and from 80 to 100 days postpartum, no significant differences were observed in protein content among cows. From 50 to 60 days postpartum and from 80 to 100 days postpartum, the fat-to-protein ratio of cows in Farm D was significantly higher than that of cows in the other three farms (p < 0.05). From 50 to 60 days postpartum, the somatic cell count of cows in Farm D (26.2 ± 66.0) was significantly higher than that in the other three farms (p < 0.05). From 80 to 100 days postpartum, the somatic cell count of cows in Farm D (36.5 ± 74.0) was significantly higher than that in the other three farms (p < 0.01). No significant differences were observed in urea nitrogen in cows from 50 to 60 days postpartum. From 80 to 100 days postpartum, the urea nitrogen of cows in Farm D (15.6 ± 2.6) was significantly higher than that in the other three farms (p < 0.01) (Table 6).

**Table 6:** Milk composition of dairy cows in four farms.

Milk composition	Time/d	Farm A	Farm B	Farm C	Farm D
Protein, %	50-60	3.20 ± 0.25	3.18 ± 0.21	3.28 ± 0.33	3.20 ± 0.31
	80-100	3.15 ± 0.26	3.19 ± 0.27	3.39 ± 0.34	3.22 ± 0.33
Fat-to-protein ratio, %	50-60	1.15 ± 0.22ᵃ	1.16 ± 0.31ᵃ	0.82 ± 0.21ᵇ	1.24 ± 0.30ᶜ
	80-100	1.18 ± 0.23ᵃ	1.14 ± 0.3ᵃ	0.85 ± 0.17ᵇ	1.27 ± 0.29ᶜ
Somatic cell count, cells/μL	50-60	11.5 ± 28.1ᵃ	13.5 ± 34.1	12.3 ± 51.8ᵃ	26.2 ± 66.0ᵇ
	80-100	10.6 ± 32.0ᴬᵃ	23.2 ± 53.6ᴬᵇ	15.7 ± 28.5ᴬ	36.5 ± 74.0ᴮ
Urea nitrogen, mg/dL	50-60	11.1 ± 1.2	10.6 ± 1.8	10.8 ± 2.4	13.2 ± 2.2
	80-100	10.8 ± 1.9	9.7 ± 2.1ᵃ	11.3 ± 2.2ᵇ	15.6 ± 2.6ᶜ

In the same row, significant differences (p < 0.05) are indicated by different lowercase letters, and extremely significant differences (p < 0.01) are indicated by different capital letters. n = 20 per farm at each time point.

### Effects of different PG health program on disease incidence of dairy cows in four farms

**Figure 2:**
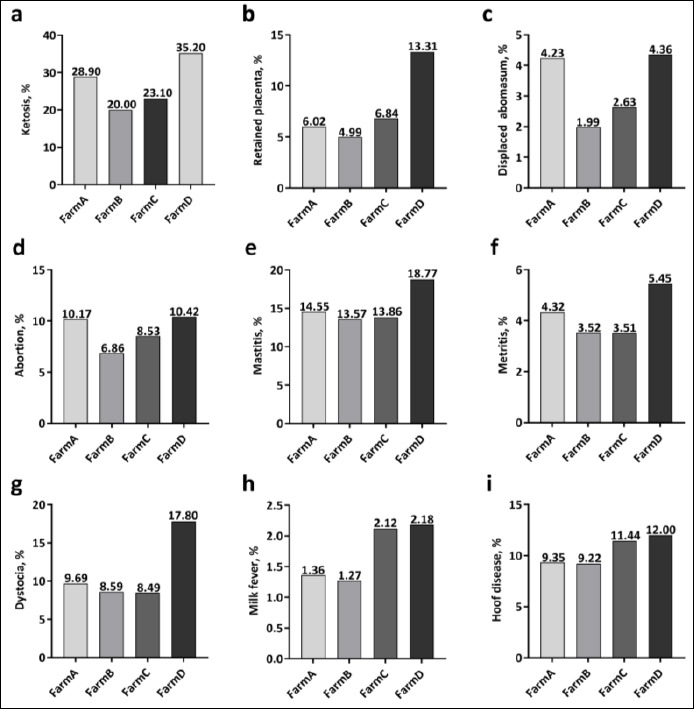
Effects of different PG health program on disease incidence of dairy cows in four farms. (a) Ketosis, (b) retained placenta, (c) displaced abomasum, (d) abortion, (e) mastitis, (f) metritis, (g) dystocia, (h) milk fever, (i) hoof disease.

Among all monitored indicators, including ketosis, retained placenta, displaced abomasum, abortion, mastitis, metritis, dystocia, milk fever, and hoof disease, the incidence rate in cows in Farm D was significantly higher than that in the other three farms (Table 7, Figures 2a-i). Notably, the incidence rates of various diseases in cows in Farm A and Farm B were relatively low, and the incidence in cows in Farm B was slightly lower than that in cows in Farm A. Cows in Farm C had relatively high incidence rates of diseases such as retained placenta, milk fever, and hoof disease, but its overall incidence was lower than that in cows in Farm D and higher than that in cows in Farm A and Farm B (Table 7, Figures 2b, h–i).

**Table 7:** Disease incidence of dairy cows in four farms.

Item	Farm A (n = 120)	Farm B (n = 120)	Farm C (n = 120)	Farm D (n = 120)
Ketosis	28.90% (35/120)	20.00% (24/120)	23.10% (28/120)	35.20% (42/120)
Retained placenta	6.02% (7/120)	4.99% (6/120)	6.84% (8/120)	13.31% (16/120)
Displaced abomasum	4.23% (5/120)	1.99% (2/120)	2.63% (3/120)	4.36% (5/120)
Abortion	10.17% (12/120)	6.86% (8/120)	8.53% (10/120)	10.42% (13/120)
Mastitis	14.55% (17/120)	13.57% (16/120)	13.86% (17/120)	18.77% (23/120)
Metritis	4.32% (5/120)	3.52% (4/120)	3.51% (4/120)	5.45% (7/120)
Dystocia	9.69% (12/120)	8.59% (10/120)	8.49% (10/120)	17.80% (21/120)
Milk fever	1.36% (2/120)	1.27% (2/120)	2.12% (3/120)	2.18% (3/120)
Hoof disease	9.35% (11/120)	9.22% (11/120)	11.44% (14/120)	12.00% (14/120)

### Effects of different PG health program on energy metabolism of dairy cows in four farms

**Figure 3:**
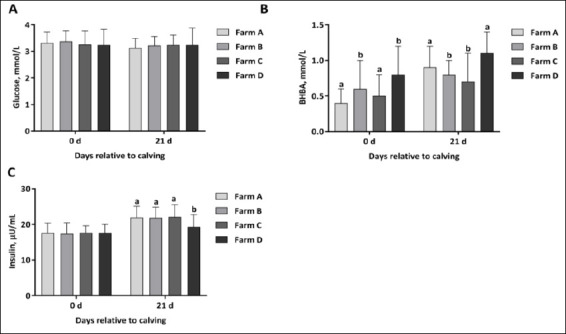
Effects of different PG health program on energy metabolism of dairy cows in four farms. n = 20 per farm at each time point. (a) Glucose, (b) BHBA, and (c) insulin. Significant differences (p < 0.05) are indicated by different lowercase letters.

On the day of calving, no significant differences were observed in glucose and insulin concentrations in cows across the four farms, and BHBA concentrations in cows from Farm B (0.6 ± 0.4) and Farm D (0.8 ± 0.4) were significantly higher than those in cows from Farm A (0.4 ± 0.2) and Farm C (0.5 ± 0.3) (p < 0.05) (Table 8, Figures 3a-c). On 21 days postpartum, there were no significant differences in glucose concentrations in cows among the four farms, and BHBA concentrations in cows from Farm B (0.8 ± 0.2) and Farm C (0.7 ± 0.4) were significantly lower than those in the other two farms (p < 0.05) (Figures 3a and b). The insulin concentration in cows from Farm D (19.31 ± 3.42) was significantly lower than that in the other three farms (p < 0.05) (Table 8, Figure 3c).

**Table 8:** Energy metabolism of dairy cows in four farms.

Item	Time (d)	Farm A	Farm B	Farm C	Farm D
Glucose (mmol/L)	0	3.31 ± 0.42	3.38 ± 0.39	3.26 ± 0.51	3.24 ± 0.58
	21	3.12 ± 0.37	3.22 ± 0.34	3.24 ± 0.37	3.24 ± 0.64
BHBA (mmol/L)	0	0.4 ± 0.2ᵃ	0.6 ± 0.4ᵇ	0.5 ± 0.3ᵃ	0.8 ± 0.4ᵇ
	21	0.9 ± 0.3ᵃ	0.8 ± 0.2ᵇ	0.7 ± 0.4ᵇ	1.1 ± 0.3ᵃ
Insulin (μU/mL)	0	17.53 ± 2.85	17.35 ± 3.10	17.62 ± 1.99	17.55 ± 2.46
	21	21.96 ± 3.12ᵃ	21.85 ± 2.98ᵃ	22.12 ± 3.45ᵃ	19.31 ± 3.42ᵇ

Significant differences (p < 0.05) are indicated by different lowercase letters.

### Effects of different PG health program on biochemical indicators of dairy cows in four farms

**Figure 4:**
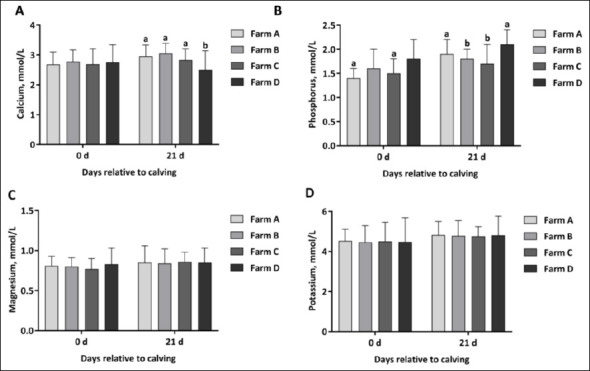
Effects of different PG health program on biochemical indicators in dairy cows. n = 20 per farm at each time point. (a) Calcium, (b) phosphorus, (c) magnesium, (d) potassium. Significant differences (p < 0.05) are indicated by different lowercase letters.

On the day of calving, no significant differences were observed in calcium, magnesium, and potassium concentrations in cows among the four farms (Table 9, Figures 4a, c, and d). However, the phosphorus concentrations in cows in Farm A (1.4 ± 0.2) and Farm C (1.5 ± 0.3) were significantly lower than those in the other two farms (p < 0.05) (Table 9, Figure 4b). At 21 days postpartum, there was no significant difference in magnesium and potassium concentrations in cows among the four farms (Table 9, Figures 4c and 4d). The calcium concentration in cows in Farm D (2.50 ± 0.64) was significantly lower than that in the other three farms (p < 0.05), whereas at 21 days postpartum no significant difference in calcium concentration was observed among cows in Farm A, Farm B, and Farm C (Table 9, Figure 4a). The phosphorus concentrations in cows in Farm B (1.8 ± 0.2) and Farm C (1.7 ± 0.4) were significantly lower than those in the other two farms (p < 0.05) (Table 9, Figure 4b).

**Table 9:** Biochemical indicators of dairy cows in four farms.

Item	Time (d)	Farm A	Farm B	Farm C	Farm D
Ca (mmol/L)	0	2.68 ± 0.42	2.78 ± 0.39	2.69 ± 0.51	2.76 ± 0.58
	21	2.96 ± 0.37ᵃ	3.05 ± 0.34ᵃ	2.83 ± 0.37ᵃ	2.50 ± 0.64ᵇ
P (mmol/L)	0	1.4 ± 0.2ᵃ	1.6 ± 0.4	1.5 ± 0.3ᵃ	1.8 ± 0.4ᵇ
	21	1.9 ± 0.3ᵃ	1.8 ± 0.2ᵇ	1.7 ± 0.4ᵇ	2.1 ± 0.3ᵃ
Mg (mmol/L)	0	0.81 ± 0.12	0.80 ± 0.11	0.77 ± 0.13	0.83 ± 0.20
	21	0.85 ± 0.21	0.84 ± 0.18	0.86 ± 0.12	0.85 ± 0.18
K (mmol/L)	0	4.53 ± 0.58	4.46 ± 0.83	4.51 ± 0.95	4.48 ± 1.21
	21	4.82 ± 0.68	4.78 ± 0.76	4.76 ± 0.48	4.81 ± 0.95

Significant differences (p < 0.05) are indicated by different lowercase letters.

### Effects of different PG health program on reproduction performances of dairy cows in four farms

The number of matings in cows in Farm B was lower than that in the other three farms, but the difference was not significant. The calving to first service interval in cows in Farm A was shorter than that in the other three farms, but there was no significant difference. The calving to first estrus interval in cows in Farm D was significantly higher than that in the other three farms (p < 0.01), whereas that in cows in Farm B was the lowest but did not differ significantly from that in the other two farms. The calving interval in cows in Farm D (406.10 ± 45.79) was significantly longer than that in the other three farms (p < 0.05), whereas that in cows in Farm B (381.83 ± 33.83) was the lowest but did not differ significantly from that in the other two farms. The conception rate in cows in Farm A (82.1) and Farm B (80.0) was significantly higher than that in cows in Farm D (73.2) (p < 0.05) and was also higher than that in cows in Farm C (78.3), although the latter difference was not significant (Table 10).

**Table 10:** Reproduction performance of dairy cows in four farms.

Item	Farm A	Farm B	Farm C	Farm D
Number of matings, time	2.53 ± 1.64	2.38 ± 1.85	2.56 ± 2.23	2.50 ± 1.56
Calving to first service interval, d	65.8 ± 5.6	66.3 ± 4.2	67.3 ± 4.8	68.2 ± 5.1
Calving to first estrus interval, d	63.85 ± 8.92ᴮ	62.91 ± 9.09ᴮ	64.58 ± 9.10ᴮ	66.01 ± 9.31ᴬ
Calving interval, d	385.64 ± 40.91ᵃ	381.83 ± 33.83ᵃ	388.65 ± 39.40ᵃ	406.10 ± 45.79ᵇ
Conception rate, %	82.1ᵃ	80.0ᵃ	78.3	73.2ᵇ

In the same row, significant differences (p < 0.05) are indicated by different lowercase letters, and extremely significant differences (p < 0.01) are indicated by different capital letters.

### Effects of different PG health program on economic benefits of dairy cows in four farms

Cows were fed PG at ¥15 per dose, and the average lactation output of diseased and healthy cows during the first 100 days postpartum was obtained from each farm and is shown in Table 11. The daily rearing costs included expenses for feed, treatment, and breeding. The average daily cost per cow among the four farms was ¥68, with a milk price of 3.21 ¥/kg. Based on the incidence of ketosis in the four farms, the economic benefits of ketotic and healthy cows were calculated for each farm.

**Table 11:** Average milk yield from the day of calving to 100 days postpartum of dairy cows in four farms.

Item	Farm A		Farm B		Farm C		Farm D	
	Ketosis	Health	Ketosis	Health	Ketosis	Health	Ketosis	Health
Average milk yield, kg/d	38.78	48.94	37.84	46.01	35.84	43.84	34.49	43.48

The economic benefit of cows with ketosis was highest in Farm D (¥59262.72) and second highest in Farm A (¥35233.15). The economic benefit of healthy cows was highest in Farm B (¥133970.40) and second highest in Farm A (¥132190.54). The total economic benefit of cows on the farm was highest in Farm A (¥166823.69) (Table 12).

**Table 12:** Economic benefits of dairy cows in four farms.

Item	Farm A		Farm B		Farm C		Farm D	
	Ketosis	Health	Ketosis	Health	Ketosis	Health	Ketosis	Health
Benefits, ¥	35233.15	132190.54	23198.40	133970.40	23883.55	118266.05	59262.72	68318.64
Total, ¥	166823.69		156868.80		141549.60		125581.36	

## DISCUSSION

### BCS, milk yield, and health outcomes

During the transition period, dairy cows undergo significant physiological changes. Due to insufficient energy intake, fat is excessively mobilized in the body, and triglycerides in the liver are further converted into ketone bodies, triggering ketosis, which has adverse effects on the health, milk yield, and reproductive performance of dairy cows. This study compared health management programs at different stages to identify the most effective program, thereby providing a scientific basis for feeding management and health care in dairy cows. The aim was to improve BCS, reduce disease incidence, and thereby increase economic benefits.

The optimal BCS for lactating cows ranges between 3.25 and 3.50, at which productive performance is in a favorable state. If BCS exceeds or falls below this range, the risk of postpartum diseases increases [36, 37]. When BCS is below the optimal range, it is more likely to cause NEB, which reduces production performance [38, 39]. At the same time, when BCS exceeds the optimal range, it can lead to obesity, and cows become prone to diseases such as fatty liver, ovarian cysts, and dystocia, while milk yield is reduced [40, 41]. Supplementing cows with PG during the transition period can reduce fat mobilization, lower NEFA concentrations, maintain glucose supply to the body, sustain milk yield while stabilizing BCS, and prevent excessive postpartum BCS loss [42, 43]. In this study, there were differences in BCS among farms. After implementing different PG health programs, the BCS of cows in Farm A was more in line with the standard range (3.25–3.50) and was associated with better lactation and productivity. Cows in Farm B had low BCS, whereas cows in Farm C had high BCS, both of which increased the incidence of diseases such as ketosis and reduced lactation. The BCS of cows in Farm D was similar to that of cows in Farm A, approaching the standard level. Although the supplemental PG content in Farm D was higher than that in Farm A, excessive body fat led to stronger fat mobilization, resulting in reduced milk yield. The milk yield levels of cows in the four farms, from highest to lowest, were Farm A > Farm B > Farm C > Farm D. This indicates that both excessive and insufficient body condition can have adverse effects on milk yield, and that reasonable supplementation with PG can mitigate these effects.

### Disease incidence and ketosis control

Excessive fat mobilization and excessive postpartum body condition decline in dairy cows can both trigger ketosis and lead to performance decline [44, 45]. NEB is also an important cause of the high incidence of postpartum diseases [46, 47], and when it occurs, it causes abnormal liver function, weakens antioxidant capacity, and triggers fatty liver, which further exacerbates the NEB state [48, 49]. PG, as a precursor of gluconeogenesis, can alleviate NEB, inhibit ketone synthesis, reduce oxidative stress, and thereby lower disease incidence [50]. Among the four dairy farms in this trial, cows in Farm D had the highest disease incidence compared with the other three farms, with the highest incidence of ketosis exceeding 35%, whereas cows in Farm B had the lowest incidence of ketosis, approximately 20%. Furthermore, the ketosis incidence rate in cows in Farm A decreased to 28.9%. Although this value was higher than the ~7% reported in a randomized controlled trial (RCT) using PG administration on days 0, 7, and 14 (500 mL of PG), the present PG health program still effectively reduced ketosis risk [51]. Similarly, our treatment effect aligns with the trend reported by McArt et al. [20, 52].

However, the primary reason for the higher ketosis incidence in Farm D was the greater BCS loss among its cows during the 7–21 days postpartum period, coupled with NEB, both of which collectively contributed to the elevated disease incidence. Consequently, even when cows in Farm D were fed high levels of PG, their ketosis incidence did not decrease, indicating that the PG supply method was inappropriate and failed to achieve the desired mitigating effect. On the other hand, prolonged or high-dose use of PG may potentially suppress the appetite of dairy cows, which might be one of the factors contributing to the failure of the PG health program in Farm D [53].

### Metabolic responses and endocrine regulation

Adiponectin is an adipokine that increases insulin sensitivity [54]. During parturition, glucose stimulates the release of insulin from the pancreas, and the levels of the two are correlated [55]. Cows with high BCS have reduced adiponectin secretion, which leads to decreased insulin sensitivity and tissue reactivity, and reduced tissue glucose uptake [56, 57]. In contrast, the use of PG can cause the body to produce more propionic acid, which is an important precursor for glucose production, thereby increasing plasma glucose concentration [58]. At the same time, PG also alleviates insulin resistance by lowering NEB, reducing fat mobilization, and maintaining a certain balance between glucose and insulin levels [59, 60]. The investigation showed that the BHBA levels of cows in Farm D were significantly higher than those in the other three farms, especially during the 7–21 days postpartum period, exceeding 1.0 mmol/L, which is consistent with the high prevalence of ketosis in cows in Farm D. After supplementing Farm D with high levels of PG, its blood glucose levels were not significantly different from those of the other three farms, indicating that PG played a role in raising blood glucose levels. However, high BHBA levels as well as low insulin levels indicated that ketosis was not being effectively treated in cows in Farm D, suggesting adverse effects of high postpartum BCS loss in these cows.

### Reproductive performance and NEB

The estrus number, estrus rate, and conception rate of cows with NEB are significantly lower than those of normal cows [61, 62]. Severe NEB can disrupt reproductive hormone secretion, leading to decreases in estradiol (E2) and progesterone (P4) levels in dairy cows, which in turn reduce reproductive performance [63–65]. Impaired reproductive performance in cows can prolong the production cycle and reduce calving efficiency [66, 67], which causes substantial economic losses to the farm [68]. In this study, cows in Farm A had the best reproductive performance, whereas cows in Farm D had poorer reproductive performance and higher disease incidence than those in the other farms, suggesting that overfeeding of PG could not fully reverse the negative effects of high postnatal body condition loss and NEB in Farm D, which resulted in reduced reproductive performance.

### Economic implications of PG health programs

The economic benefit of the different farms was mainly composed of morbid and healthy cows, with morbid cows having lower average economic efficiency; therefore, cow morbidity had a major impact on the economic benefit observed in this study. Due to the high incidence of ketosis in cows in Farm D, the economic benefit of morbid cows accounted for approximately 47.2% of the overall economic benefit, which was much higher than that of the other farms during the same period, and was the main reason for the low economic benefit of cows in Farm D. The analysis of economic benefits showed that cows in Farm A had the highest milk production and a relatively low overall disease incidence rate, thus achieving the highest economic benefits. While the economic analysis provides a practical comparison among farms, it was relatively simplified. Labor and veterinary costs associated with PG drenching were not included, and only the benefits from improved production and reduced disease risk were considered. This represents a limitation of the present study. Furthermore, to verify the robustness of the economic results, a sensitivity analysis was performed based on key parameters (milk price, daily cost per cow, and milk yield), with each parameter varying by ±5% and ±10%. The results showed that the economic trends among farms remained stable, confirming the reliability of the findings.

### Practical implications of PG programs

In this study, although Farm B performed well in terms of disease incidence and metabolic parameters, this might be attributed to universal preventive PG administration to all cows on the day of calving. In contrast, Farm A adopted a targeted, diagnosis-based treatment approach: 500 mL of PG was administered for 2 consecutive days at 7 and 14 days postpartum only to cows diagnosed with ketosis. This approach ensured effective intervention during the peak ketosis risk period (3–14 days postpartum), while avoiding excessive supplementation in healthy animals. The overall effect of Farm A’s health program was significantly better than that of Farm C and Farm D, which could be attributed to the targeted and continuous PG supplementation following ketosis diagnosis. Notably, compared with the other three farms, Farm A achieved better body condition, higher milk yield, and greater economic benefits, indicating that this strategy is more practical and efficient under commercial farm conditions. Therefore, the outcomes from Farm B do not alter the conclusion that Farm A’s health program yielded the best overall performance.

## CONCLUSION

The present study demonstrated that different PG-based health programs exerted distinct effects on BCS, milk yield, metabolic status, disease incidence, reproductive performance, and economic outcomes in dairy cows during the transition period. Among the four farms evaluated, cows in Farm A exhibited BCS within the optimal range, higher milk yield, improved metabolic stability as indicated by lower BHBA concentrations, reduced disease incidence, better reproductive performance, and the highest overall economic benefit. In contrast, cows in Farm D showed higher BHBA concentrations, greater disease incidence, poorer reproductive performance, and reduced economic returns, despite receiving higher PG supplementation, indicating that excessive or improperly timed PG administration does not guarantee improved outcomes.

From a practical perspective, the findings highlight that targeted, diagnosis-based PG administration is more effective than blanket or excessive supplementation strategies. Administering PG during the critical postpartum window (7–14 days) to cows diagnosed with ketosis optimizes energy balance, stabilizes BCS, and enhances productivity while avoiding unnecessary treatment in healthy animals. These results provide a practical and economically viable framework for improving transition cow management under commercial farm conditions.

A major strength of this study is the multi-farm comparative design under real-world conditions, integrating production, metabolic, clinical, and economic indicators within a single framework. This approach enhances the applicability of the findings for large-scale dairy operations. However, several limitations should be acknowledged. The observational nature of the study and the absence of a non-PG control group limit causal inference. In addition, economic analysis did not include labor and veterinary costs associated with PG administration, and management differences among farms could not be fully standardized.

Future research should focus on controlled multi-farm trials to validate these findings, optimize PG dosage and timing strategies, and investigate long-term impacts on herd health and productivity. Further studies integrating precision feeding, metabolic monitoring, and individualized treatment approaches may provide deeper insights into improving transition cow management.

In conclusion, an appropriately timed and targeted PG health program, as implemented in Farm A, represents the most effective strategy for improving metabolic health, productivity, and economic efficiency in dairy cows during the transition period.

## DATA AVAILABILITY

The data that support the study are available from the corresponding author upon reasonable request.

## AUTHORS’ CONTRIBUTIONS

CX and YXS: Conceived and designed the study, developed the methodology, and provided project coordination. XZY: Performed the experiments, conducted data collection, and carried out laboratory work. XZY, GS, and SYH: Performed data analysis, statistical interpretation, and data curation. XZY: Prepared the original draft of the manuscript. LYW, XCJ, and KYY: Performed formal analysis, data visualization, and figure preparation. CX, YXS, XZY, LYW, XCJ, KYY, SYH, and GS: Performed a critical review of the manuscript and ensured compliance with ethical protocols. All authors have read and approved the final version of the manuscript.
